# Computer Simulation of Metastable Fluid States in the Lennard-Jones System

**DOI:** 10.6028/jres.080A.011

**Published:** 1976-02-01

**Authors:** Harold J. Raveché, William B. Streett

**Affiliations:** Institute for Basic Standards, National Bureau of Standards, Washington, D.C. 20234; Science Research Laboratory, U.S. Military Academy, West Point, New York 10996

**Keywords:** Glassy state, Lennard-Jones system, Monte Carlo simulation, nucleation, overcompression, supercooling

## Abstract

Using the Monte Carlo method in statistical mechanics, we have simulated high density metastable states. We find that nucleation from a three dimensional fluid array to a crystalline solid is possible, but that periodic boundary conditions and the small size of the system inhibit the formation of perfect crystals. Evidence for the existence of an amorphous solid state has also been observed, and the pair correlation function of this state exhibits some of the features associated with random close-packed arrays of hard spheres. The possible relation between these simulations and the formation of glassy states in real systems is briefly discussed.

## 1. Introduction

Thermal and structural properties of metastable fluid states associated with subcooling are of interest in several problems, including glass formation, nucleation and crystal growth, and disordered alloys.

We report briefly on Monte Carlo [1]^1^ simulations of metastable states in systems of 108 and 256 particles interacting through the Lennard-Jones 6–12 potential function. The energy and length parameters are denoted by *ϵ* and *σ*, respectively. With *N_A_* the Avogadro constant and *k_B_* the Boltzmann constant, the following reduced units are used: volume, *σ*^3^; pressure, *ϵ*/*σ*^3^; energy, *N_A_ϵ*; and temperature, *ϵk_B_*. We report simulations at temperatures of *T*=0.80, 1.17, and 2.74 for densities between 1.05 and 1.38. Pressures for these states are well into the kilobar range.

The computations proceed as follows. The coordinates of a disordered fluid array, obtained from an equilibrated simulation at a liquid-like density, are scaled to a high density at a temperature at which the lowest energy state is that of a crystalline (fcc) solid [2]. (The initial configuration thus has the character of a metastable fluid state, which can be realized in a real fluid by undercooling or overcompression.) We then determine whether the simulation converges to particular values of the pressure and internal energy, and whether, on the molecular level, it converges to a state which is not noticeably labile.

We find that the thermodynamic properties do in fact converge, after runs of the order of 10^7^ configurations, but that the resulting pressures and energies are higher than those of the crystalline solid at the same density and temperature. Examination of the molecular structure indicates that some of these runs converged to distorted fcc crystals, while others converged to comparatively amorphous states, with structures similar to the random close-packed structures studied by Finney [3] and Bernal [4]. Such structures have been discussed by Turnbull [5] as a possible model for the ideal glass state.

Although the sequence of configurations generated in a Monte Carlo simulation is produced by a random process, each configuration differs from the preceding one by at most a small displacement of a single particle. Hence it is not unreasonable to consider that the progress of the simulation bears an approximate relation to the progress of real time. Examination of the molecular structures resulting from these simulations may therefore provide some insight into crystal nucleation and glass formation in metastable fluids composed of simple molecules. Nucleation phenomena similar to those reported here have recently been observed in molecular dynamics simulations of the same model [6]. Previous work on soft-sphere systems [7] also indicates similar results for repulsive potentials which vary as the inverse twelfth power of the internuclear separation.

## 2. Results and Discussion

The results for the pressure and energy at *ρ* = 1.375 and *T*=2.74 are shown in figure 1a; this density is about 30 percent beyond the freezing density which was reported [2] for the *T*= 2.74 isotherm. The solid curves are the cumulative averages and the dashed curves are the averages over the most recent 10^5^ configurations. The breaks in the solid curve at 9 × 10^6^ and 16 × 10^6^ configurations indicate that the particle coordinates at these points were used to initiate a new simulation. The very close agreement between the solid and dashed curves beyond 17 × 10^6^ clearly indicates convergence. (The corresponding results for the internal energy behave similarly.) The nearly horizontal curves at the left of figures 1a and 2a were characterized by extensive displacements of the particles from their initial positions–in other words “liquid-like” behavior. As the pressure and energy decreased, the net particle displacements over each 10^5^ configurations likewise decreased. At the horizontal portion of the curves at the end of each run, the molecular structure was no longer labile; that is, the particles appeared to be locked into a fixed array undergoing only small displacements from their mean positions as the computation proceeded. The pressure and energy converged to values of 80 and −0.5, respectively. The pressure and energy obtained from a simulation of an fcc array at the same density and temperature are 72 and −1.9, respectively.

The pair correlation function, *g*(*r*) at 5 × 10^6^ and at 27 × 10^6^ is shown by the solid curves in figure 1b and figure 1c, respectively. For comparison, the correlation function for a simulation of an fcc array at the same density and temperature is shown by the dashed curve in figure 1c. The local maxima in this curve occur approximately at the first, second, third, etc., nearest neighbor distances of the fcc solid. The results in figures 1b and 1c suggest that nucleation has occurred and that the initially metastable fluid has converged to an imperfect crystalline array. Visual inspection of three-dimensional structure at 27 × 10^6^ configurations by means of stereoscopic pairs of 35 mm photographs confirms the existence of distorted crystalline planes.

More striking evidence for nucleation to a crystalline array was obtained at *ρ* = 1.105 and *T*= 0.80, which is less than one-half the freezing temperature [2] at this density. The results for the pressure and the average root mean square displacement of a particle (rms) from its initial position are shown in figure 2a. The pressure over the most recent 10^5^ configuration converged to 8.93 and the result for a run begun from fcc positions was 9.03. The internal energy for both runs converged to the same value, −7.17. The behavior of the rms suggests that as the system equilibrates the positions of the atoms are not particularly labile. This can be qualitatively interpreted as restricted diffusion.

The pair correlation for the runs are shown in figure 2b, as before the dashed curve is for the run begun from fcc coordinates and the solid curve is for the run begun from a random array. Both curves are identical to radial distances comparable to the second nearest-neighbor distance and the close similarity for larger interatomic separation clearly indicates crystalline order. These results should be compared with figure 2c which shows the pair correlation function at 2.0 × 10^6^ configurations in the course of the run begun from the random array. Evidently the local order in the random system underwent distinct changes between 2.0 and 20 × 10^6^ configurations.

The formation of a perfect crystalline lattice in these simulations is greatly hindered by the periodic boundary conditions which are used to avoid surface effects. Unless the crystal nucleates with its principal axes parallel to the axes of the reference coordinate system, it will necessarily undergo a severe strain at the boundaries of the cube in which the particles are confined. It therefore seems likely that homogeneous nucleation has occurred in these runs but that the failure to reach a perfect crystalline structure is an artifact of the periodic boundary conditions and perhaps of the small size of the system.

Similar computations on the *T*=2.74 isotherm were performed at *ρ*= 1.30 which is approximately 20 percent beyond the freezing density [2]. The results are given in figures 3a and 3b. The general behavior of the pressure and energy is similar to the previous simulation. (The pressure and energy after convergence are 59 and −2.4, respectively, compared to 53 and −3.3 for a simulation of an fcc array at the same temperature and density.) The molecular order, however, suggests different features. The pair correlation function in figure 3b shows no evidence of the fcc ordering indicated by the solid curve in figure 2b. Visual inspection of the atomic coordinates at 19 × 10^6^ configurations by means of stereoscopic pairs of photographs indicated an amorphous structure, with little evidence of distorted crystalline planes. During the same sequence of configurations, the molecular structure gradually ceased to be labile and the particles became locked onto a disordered array. This was also manifested in the pair correlation function which did not change by more than ± 1 percent beyond 14 × 10^6^ configurations.

We infer from this that the molecular order became locally more close-packed as the pressure and energy converged. The amorphous structure at convergence is suggestive of the formation of a glassy state. In figure 3b there is no evidence of the subdued second nearest neighbor peak at *r* ≃ 1.45, present in the solid curve of figure 2b. In previous studies [2] we have observed that the existence of fcc lattice structure results in a subdued peak or “step” in this region of the pair correlation function. The pair correlation shown in figure 3b exhibits some of the features found in studies of random close-packed arrays of hard spheres [3]. In particular, the broad second maximum can be interpreted as two separate peaks, centered at approximately *r*=1.75 and *r*=1.95. Bernal [4] attributes the first of these to the presence of tetrahedra which share a common base, and the second to the collineation of three particles.

The relation between the structure of a random close-packed system and that of an ideal glass has been discussed in detail by Turnbull [5]. It is sufficient to remark here that the structure of the glass state in macroscopic systems is still an important unresolved problem.

We have investigated the reproducibility of the simulations in the metastable region by repeating the calculations with initial coordinates obtained from a random number generator. The results for *ρ*= 1.30 and *T*= 2.74 are shown in figure 4a. These should be compared to the results shown in figure 3a which, as we have noted, are for a simulation begun with the coordinates of an equilibrated run at liquid-like densities [2]. The dashed curves indicate the simulations are reproducible to within 4 percent and we conjecture that the differences would be smaller if the simulation in figure 4a had been continued to the same number of configurations as that in figure 3a.

The pair correlation function obtained from the simulation in figure 4a at 15 × 10^6^ is quite similar to that shown in figure 3b. In particular the function shows no evidence of the crystallinity shown in figures 1b and 2b.

The constant pressure heat capacity for the Lennard-Jones system can be computed in terms of pressure and energy fluctuations, Δ*P* and Δ*U* respectively, by

$$C_{p}=\frac{\langle {\left(\Delta U\right)}^{2}\rangle }{k_{B}T}-\frac{1}{k_{B}^{2}T^{3}}\times \left[\frac{{\langle \Delta P\Delta U\rangle }^{2}}{\langle {\left(\Delta P\right)}^{2}\rangle /k_{B}T-7P/V+8U/V^{2}}\right].$$

The first term on the right is the constant volume heat capacity and the second term represents the ratio of the square of the compressibility to the expansivity. For potentials of the Lennard-Jones type (integer power law function) the ratio can be written in terms of pressure and energy fluctuations [8].

The results for the configurational heat capacity for the simulation shown in figure 4a are given in figure 4b. Since the pressure and energy fluctuations are large, the heat capacity also has large fluctuations. For reference, we note that the heat capacity of the crystalline solid was estimated to be 3.4 which is close to the result for the glassy state shown in figure 4b.

**Figure 5:**
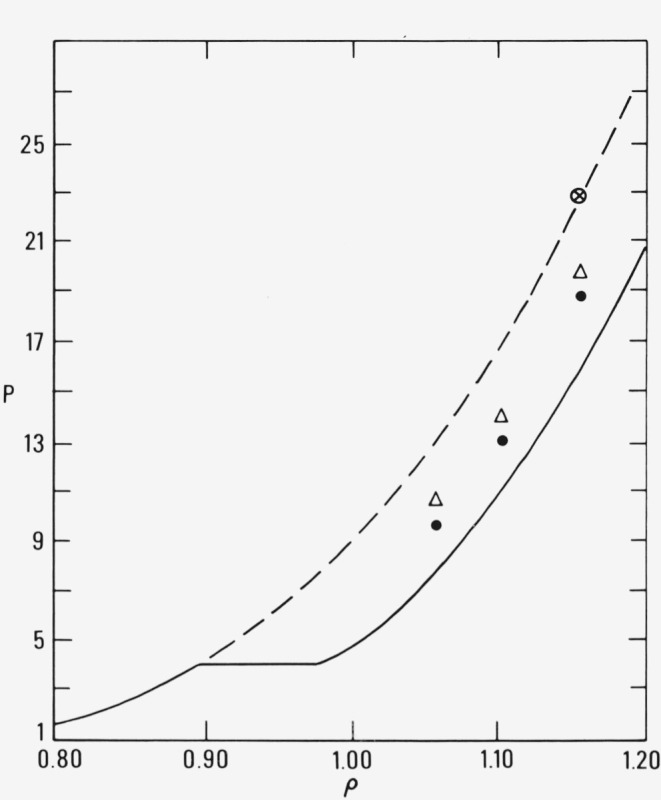
Results on the *T* = 1.17 isotherm with 108 (△) and 256 (·) particle systems. The circled crisscross denotes the density. The density was successively decreased and the coordinates of each preceding state were used to initiate a new simulation.

Size effects have been studied by performing simulations on 108 particle systems. There is one noticeable difference between the 108 and 256 particle systems: the convergence is not as complete in the smaller system. That is, the converged values of the pressure and energy in the smaller system are higher than those in the larger system at the same density and temperature. This is shown in figure 5 where we have plotted results from both the 108 (denoted by triangles) and the 256 (denoted by solid circles) particle systems on the *T*= 1.17 isotherm [2]. The circled crisscross denotes the density at which both size systems were initially prepared in a fluid array. After the systems had reached convergence in pressure and energy, the density in each system was successively decreased and the coordinates from each preceding simulation were used to begin a new run. From figure 5 we note that data points for both size systems form smooth curves which give higher pressures than those of the fcc crystalline phase. As with the results on the *T*=2.74 isotherm, the data in figure 5 show that nucleation is possible but that the periodic boundary conditions and small size prevent complete crystallization. Below densities of approximately *ρ* = 1.05, the systems “melt” and, as expected, the pressures join to the fluid branch.

In summary, we have shown that nucleation from a fluid to a crystalline array is possible in a small three dimensional system with periodic boundary conditions. The results indicate that there is an amount of overcompression or supercooling which must be exceeded to observe the characteristic decrease in pressure and internal energy. That is, unless the overcompression is of a certain magnitude, the thermodynamic properties of the initially random arrays appear to fluctuate about values which are typical of a smooth extension of the equilibrium fluid phase. For the isotherms we have investigated the lower limit of overcompression appears to be 15–20 percent. For a given density, the investigations suggest that even a larger amount of supercooling may be required. We have examined the role of size in determining the completeness of the observed crystallization. In addition, we have found evidence for the existence of an amorphous close-packed array which has some of the characteristics of a model proposed [5] for the glass state in macroscopic systems. If the existence of this state is confirmed in additional studies, it may prove useful in the study of the molecular structure of the glass state. Since we have seen that the small systems can undergo nucleation to a crystal, this suggests that the amorphous solid state may be a well defined metastable state between the fluid and crystalline phases.

The computations suggest further investigations at high densities and lower temperatures. We have preliminary results for several states which are consistent with those reported here.

## Figures and Tables

**Figure 1a:**
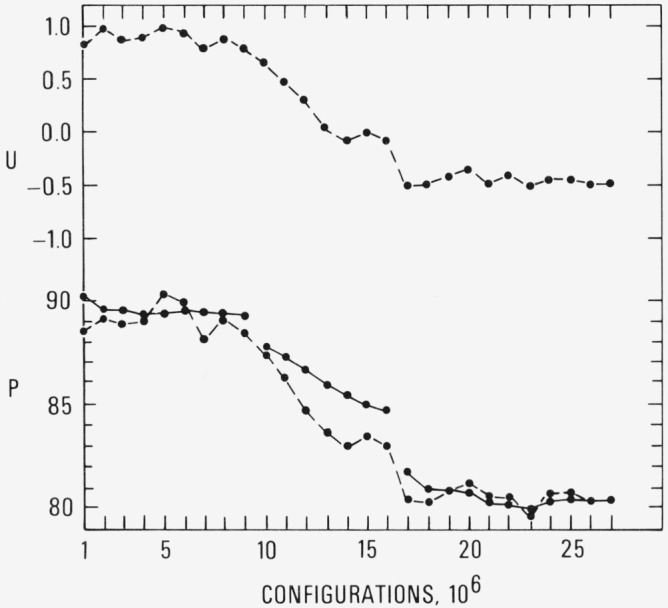
The pressure and energy (in Lennard-Jones units) as a function of the number of configurations at ρ = 1.375 and *T* = 2.74. The dashed curves are averages over the most recent 10^5^ configurations and the solid curves is the cumulative average. The breaks in the solid curve indicate that the particle coordinates at these points were used to initiate a new simulation.

**Figure 1b:**
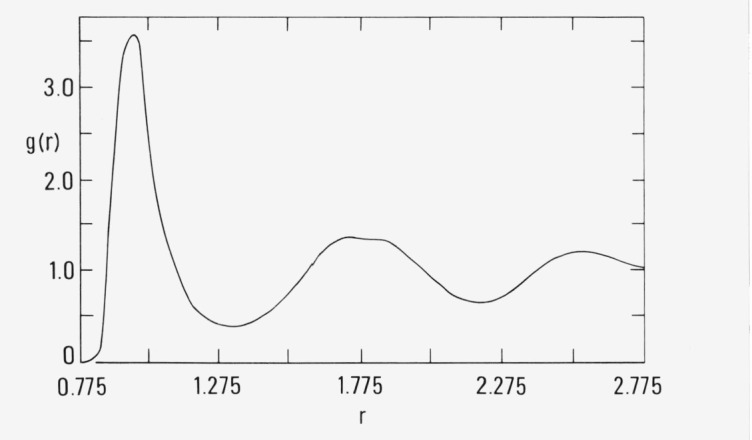
The pair correlation function, g(r), for the simulation in figure la at 5 × 10^6^ configurations.

**Figure 1c:**
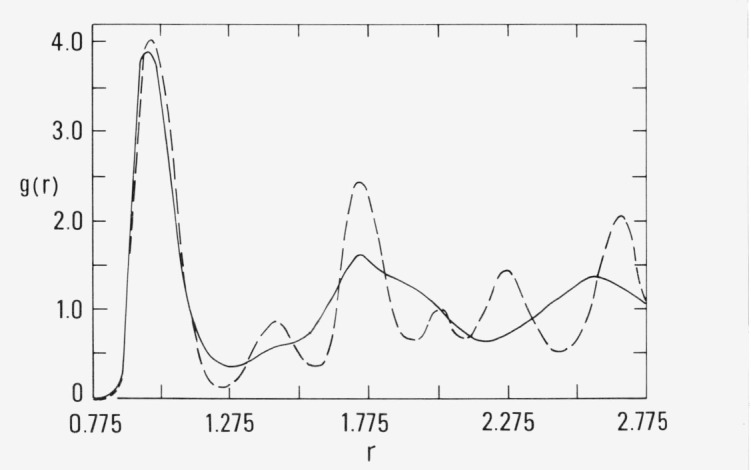
The same as figure 1b except solid curve is at 2.7 × 10^7^ configurations and the dashed curve is from the simulation begun from fcc array.

**Figure 2a:**
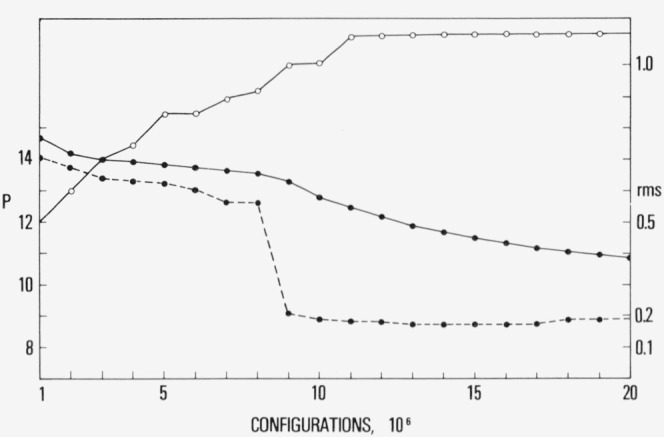
The pressure and average root mean square displacement (rms) for ρ= 1.105 and *T* = 0.80. The rms (open circles) is in units of the distance parameter, *σ*.

**Figure 2b:**
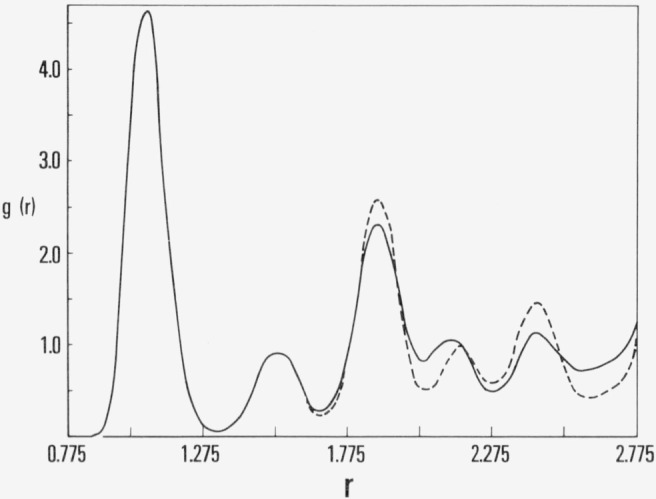
The pair correlation function, *g*(*r*), at 2.0 × 10^7^ configurations for the simulation shown in figure 2a. The result for the simulation begun from fcc array is given by dashed curve.

**Figure 2c:**
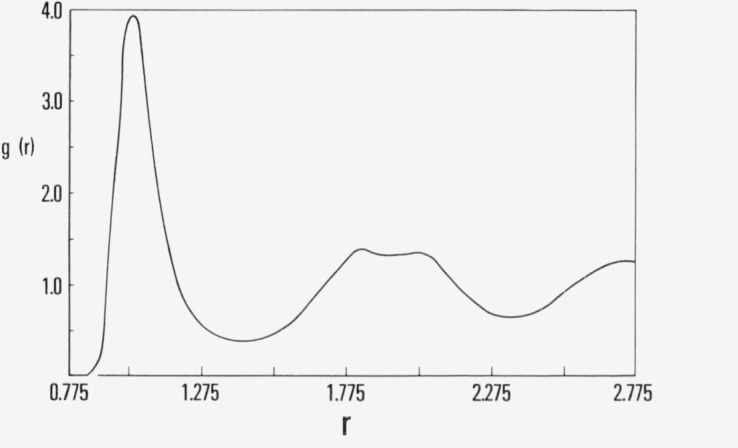
The pair correlation function at 2.0 × 10^6^ configurations for the simulation in figure 2a.

**Figure 3a:**
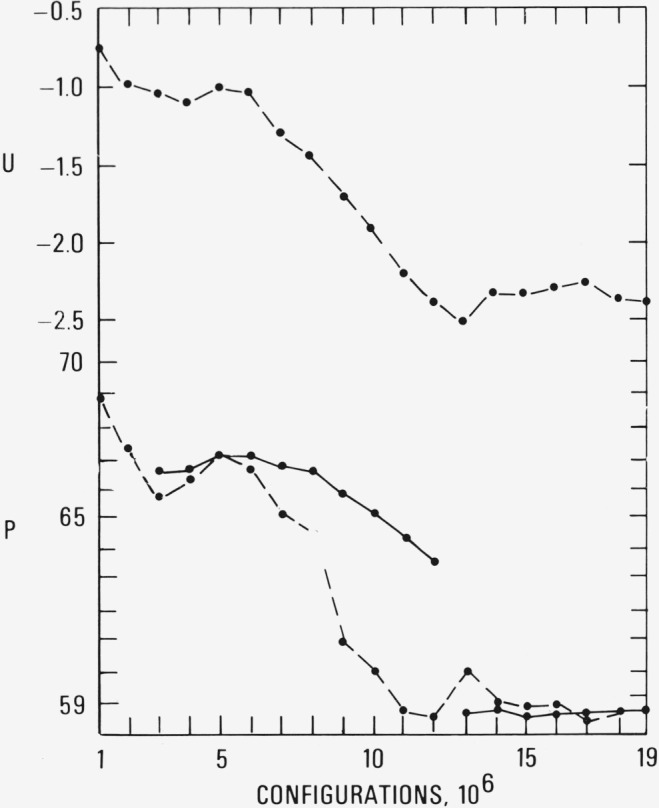
The same as figure 1 a except ρ = 1.30.

**Figure 3b:**
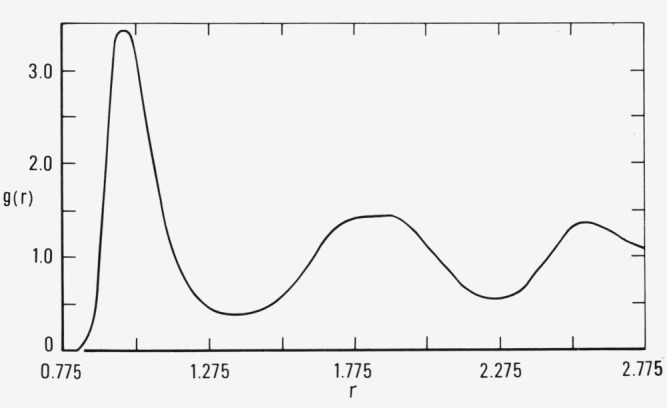
The pair correlation function, *g*(*r*), for the simulation shown in figure 2a at 1.9 × 10^7^ configurations.

**Figure 4a:**
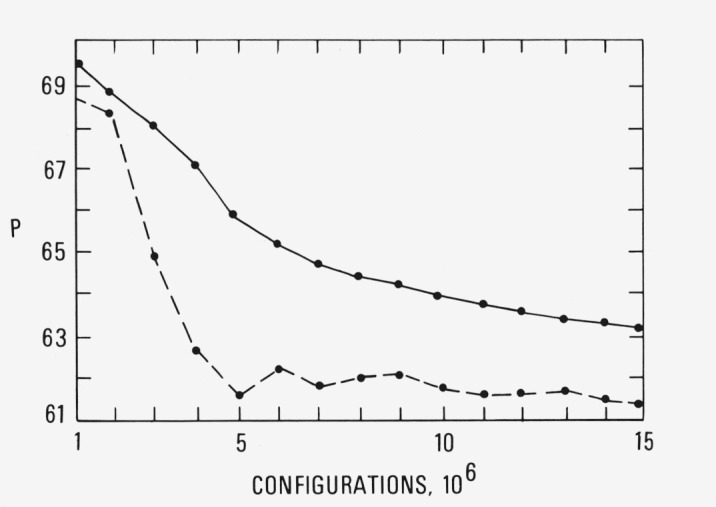
The pressure for simulation at ρ = 1.30 and *T* = 2.74 begun from coordinates obtained from a random number generator.

**Figure 4b:**
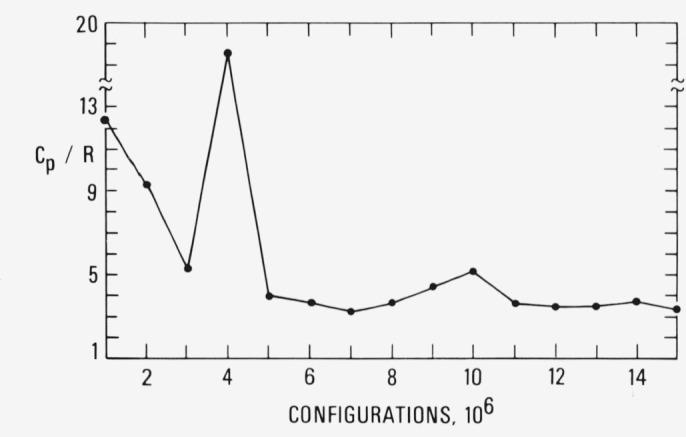
The constant pressure heat capacity in units of the gas constant for the simulation shown in figure 4a.

## Footnotes

1 Figures in brackets indicate the literature references at the end of this paper.
